# Supplementary figures and images for: Y chromosome shredding in Anopheles gambiae: Insight into the cellular dynamics of a novel synthetic sex ratio distorter
Source: PLoS Genet. 2024 Jun 7;20(6):e1011303. doi: 10.1371/journal.pgen.1011303 (PMC11189259; doi:10.1371/journal.pgen.1011303)

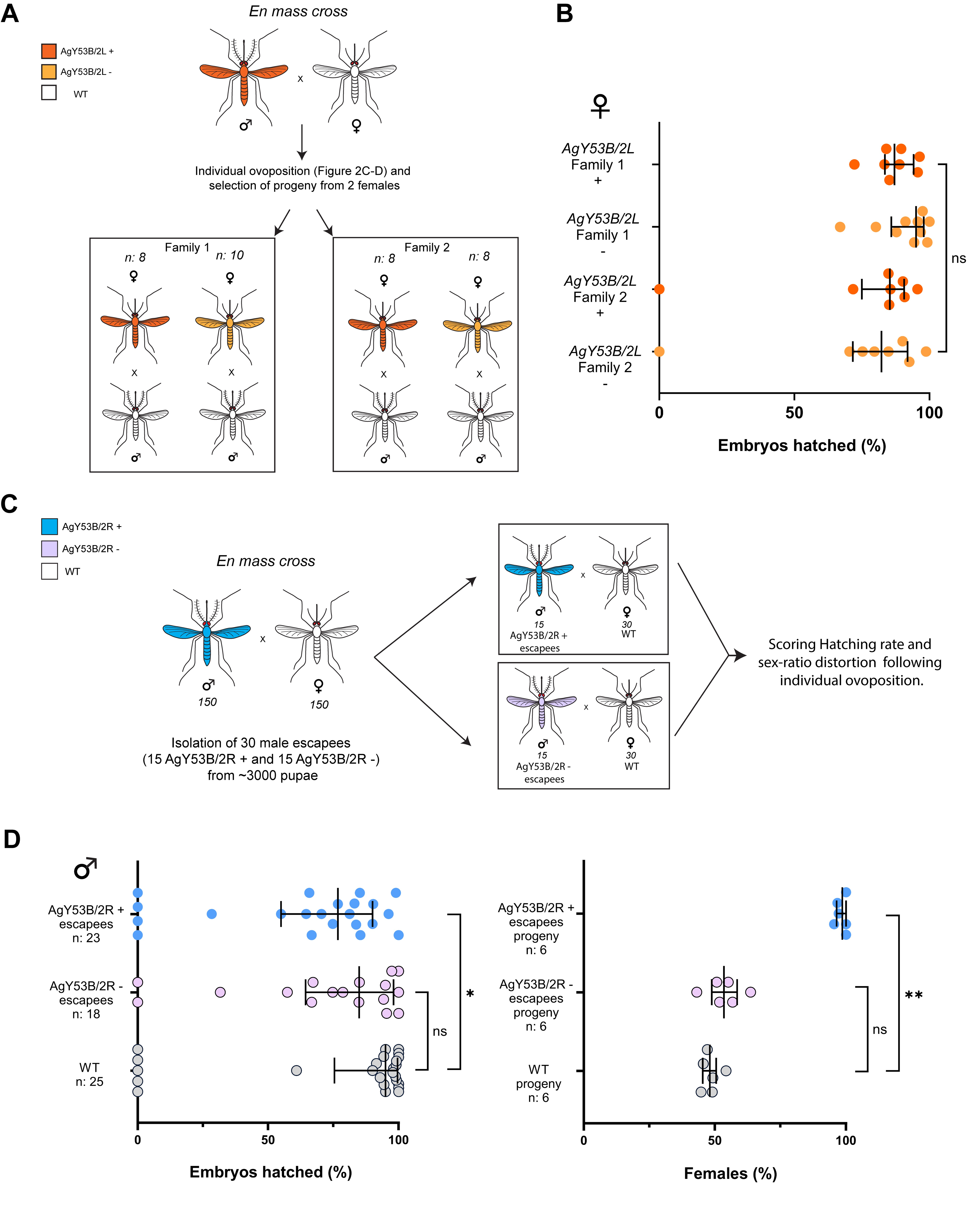

Supplement: S1 Fig — (A) Schematic of the genetic cross used to isolate two different families in the progeny of AgY53B/2L + males, this refers to the phenotypic assay shown in Fig 2C (main text). Transgenic and non-transgenic female progeny (AgY53B/2L + and AgY53B/2L -, correspondingly) were separated in different cages and crossed with 20 WT An. gambiae males. Number of females used for each cross are shown. Following blood feeding, females were allowed to lay individually and the HR was scored (B). No significant reduction in the HR was detected in AgY53B/2L + as well as in non-transgenic sibling females (Dunn’s multiple comparison test, P value > 0,05). (C) Schematic of the genetic cross used to isolate escapee males from strain AgY53B/2R. Transgenic and non-transgenic escapee males were crossed to 30 WT females in two separate cages and a phenotypic assay was performed as previously described. In addition, sex ratio for six randomly selected progenies was analysed (D). A slight, but significant, reduction in the HR (Dunn’s multiple comparison test, P value = 0.0448) was observed when comparing AgY53B/2R + escapee males with the WT control. The analyses of the progenies randomly selected shows high female bias for AgY53B/2R + males and value in line with Mendelian segregation for AgY53B/2R –and WT controls. Median and interquartile ranges are shown. (TIF) [file pgen.1011303.s002.tif]

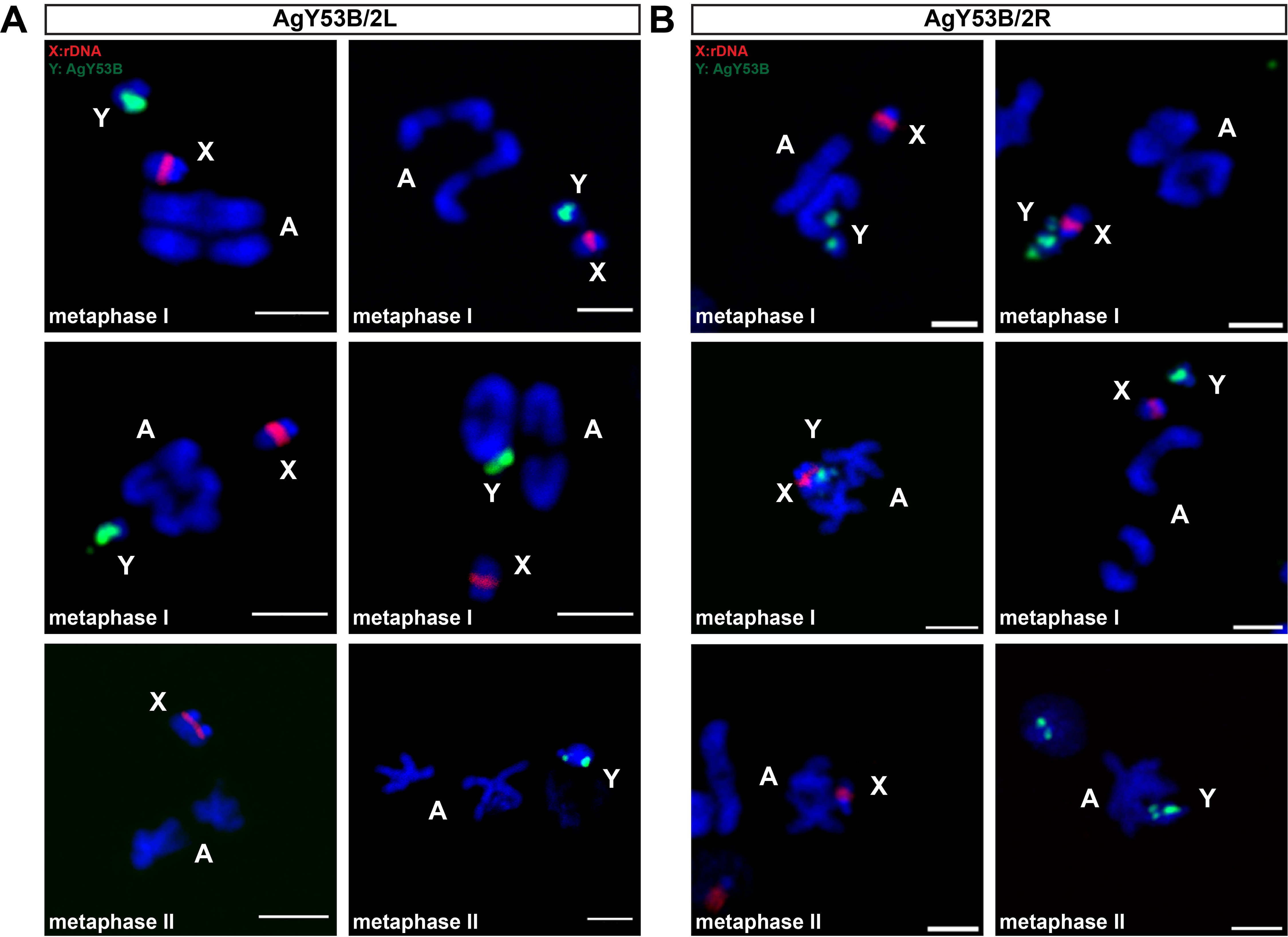

Supplement: S2 Fig — (A) Both sex chromosomes are detectable in metaphase I of cells obtained from the testis of AgY53B/2L strain. Chromosomes in metaphase plate and chiasmata can also be detected. Cells in Metaphase II bearing X or Y chromosome can also be observed indicating that correct segregation of sex chromosomes can still occur despite Y-chromosome lagging. (B) A similar scenario can also be observed in the strain AgY53B/2R where metaphase I and II cells show the presence of both sex chromosomes. For comparison with metaphase karyotype from WT strain see Liang and Sharakhov, 2019 (15). Scale Bar = 3 μm. Blue = DAPI, Red = X chromosome-specific probes (X: rDNA), Green Y chromosome-specific probe (Y: AgY53B). (TIF) [file pgen.1011303.s003.tif]

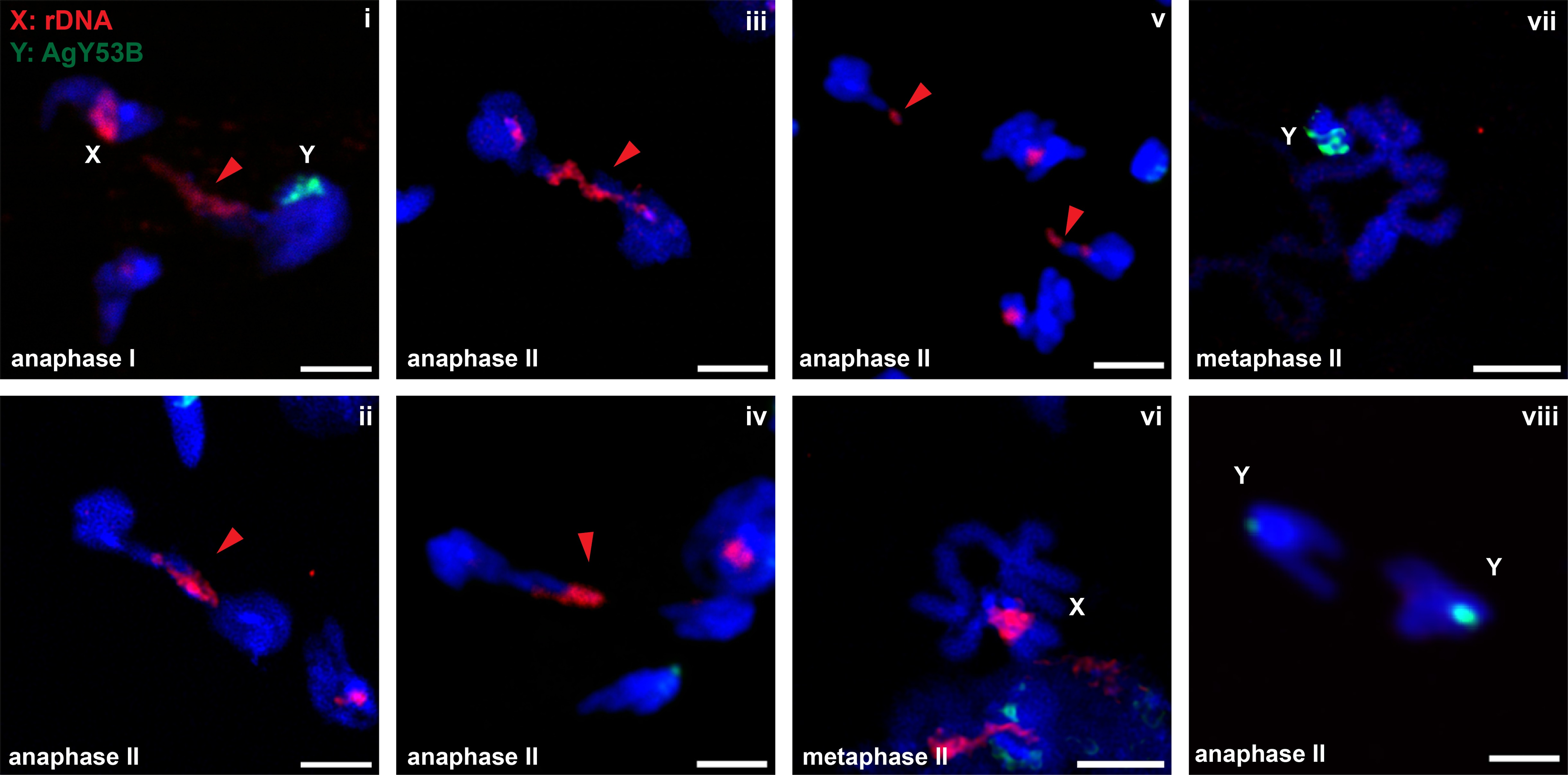

Supplement: S3 Fig — DNA FISH on meiotic chromosomes obtained from Ag(PMB)1 testis. Panel i shows X-chromosome lagging during anaphase I, while Y chromosome segregate at the pole of the cell. Panels ii-iii-iv-v show X-chromosome lagging during anaphase II. Panels vi and vii show sex chromosomes metaphase II. Panel viii shows cells in anaphase II with no lagging of the Y chromosome. Red = X chromosome specific probes (X: rDNA). Green = Y chromosome-specific probe (Y: AgY53B). Scale bar 3 μm. red arrowheads the lagging chromosome. (TIF) [file pgen.1011303.s004.tif]

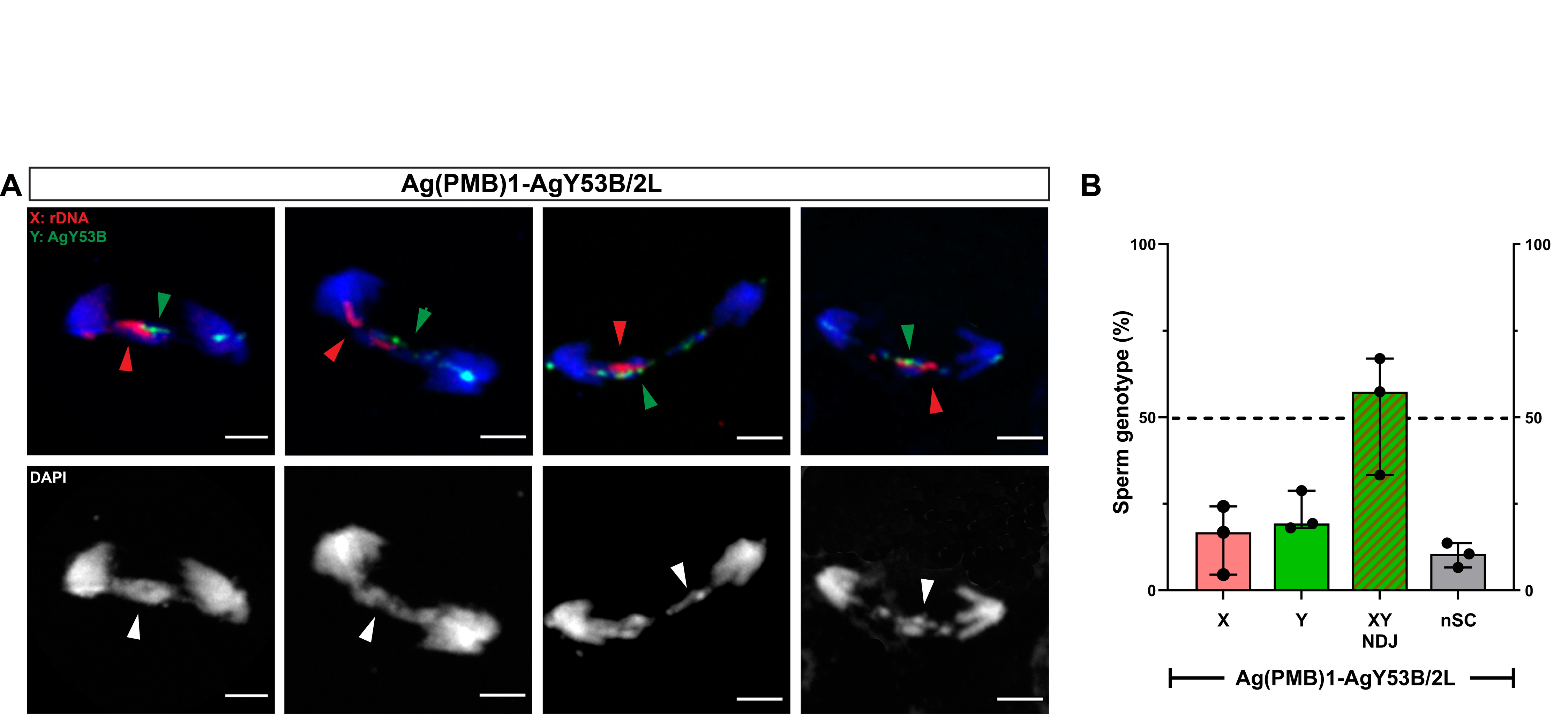

Supplement: S4 Fig — (A) DNA FISH reveal lagging sex chromosomes during meiotic anaphase I in trans heterozygous Ag(PMB)1-AgY53B/2L males. The lagging pattern is similar to the one detected in trans heterozygous Ag(PMB)1-AgY53B/2R shown in Fig 5C (main text). Red and green arrowheads indicate lagging sex chromosomes. Red = X chromosome-specific probes (X: rDNA). Green = Y chromosome-specific probe (Y: AgY53B). Scale bars = 3 μm. (B) Sperm sex chromosome genotypes of testes obtained from 3 trans heterozygous Ag(PMB)1-AgY53B/2L males. Median of the percentage of X-bearing = 16.75%, Y-bearing = 19.29%, XY-NDJ = 57.36%, nSC = 10.53%. We observed a higher proportion of XY NDJ in testis dissected from Ag(PMB)1-AgY53B/2L compared to Ag(PMB)1-AgY53B/2R males (see Fig 5D, median XY NDJ of the strain Ag(PMB)1-AgY53B/2R = 19.65%). Total number of sperm counts: X = 55, Y = 81, XY NDJ = 224, nSC = 36. In each bar median with Standard Deviation (SD) is shown. (TIF) [file pgen.1011303.s005.tif]

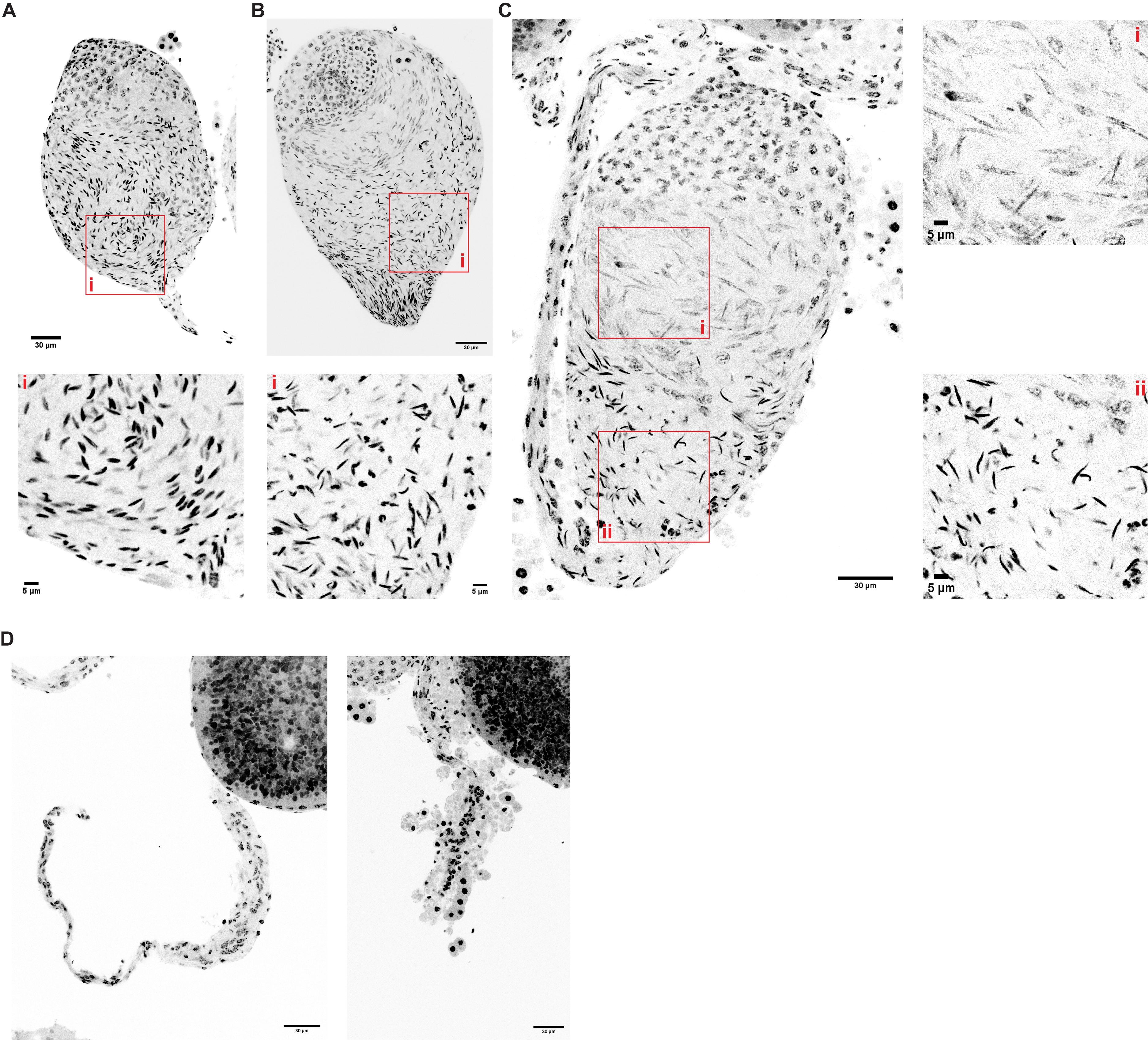

Supplement: S5 Fig — (A) Control sibling males harbouring no genetic construct. We observed sperm nuclei showing normal arrow-like shape (B) Testis dissected from sibling males harbouring only CRISPRAgY53B construct (AgY53B/2R). In these males some sperm show chromatin condensation defects. This can be linked to the presence of defective sperm with X-Y NDJ according to the previous analyses on sperm sex chromosome genotype. (C) Testis dissected from trans heterozygous Vasa2:Cas9-AgY53B/2R males. Spermatids and mature sperm show evident chromatin condensation defects, with elongated and irregular shapes (C-i-ii). Nevertheless, some sperm show a normal-like shape (C-ii). (D) Atrophic-like testes dissected from trans heterozygous Vasa2:Cas9-AgY53B/2R males. In these testes no mature sperm were detected. These testes have a smaller size and contains a lower number of cells if compared to the testes in A, B and C. All the testes samples were dissected from one day old adult males. At least 4 testes for each group were analysed. (TIF) [file pgen.1011303.s006.tif]

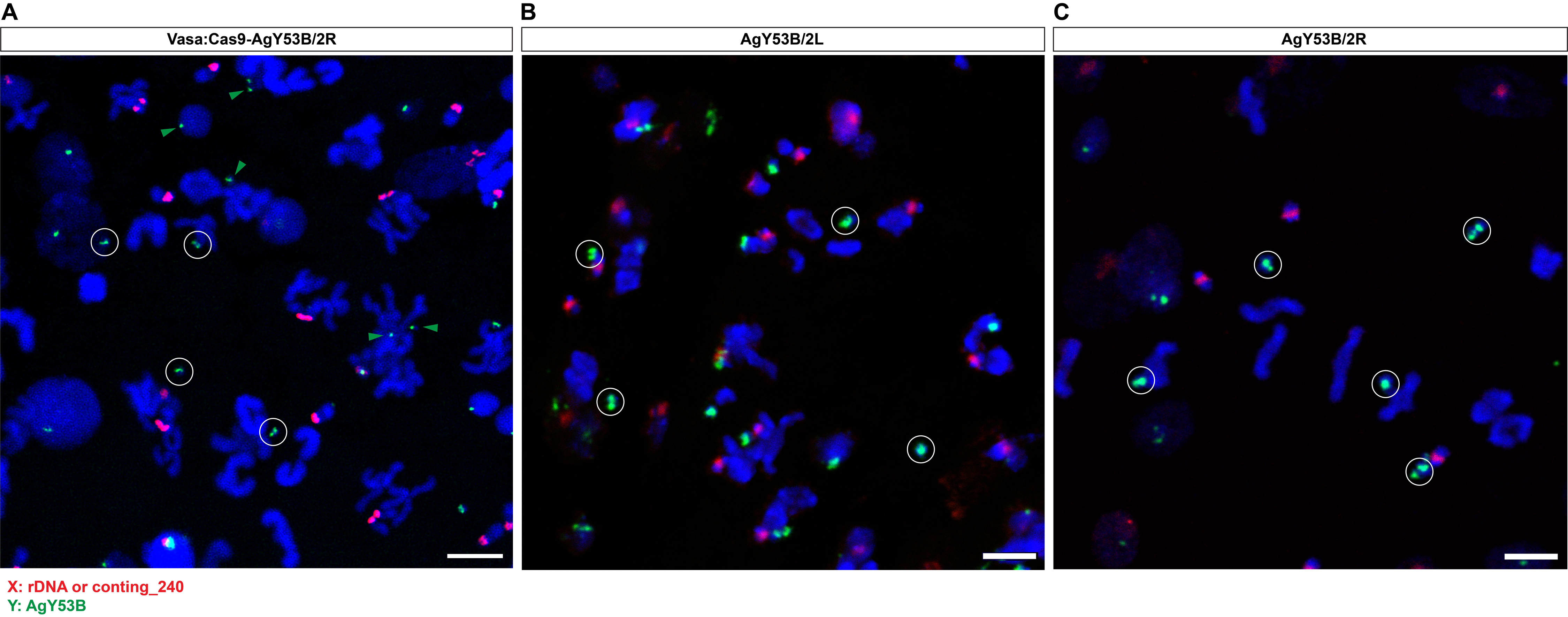

Supplement: S6 Fig — DNA FISH on meiotic chromosomes spreads obtained from Vasa:Cas9-AgY53/2R trans heterozygous males (A), AgY53B/2L (B) and AgY53B/2R (C) strains. (A) Pre-meiotic shredding of the Y chromosomes is achieved using Vasa2 promoter in combination with CRISPRAgY53B construct present in the strain AgY53B/2R. Signals from target site specific probe reveal the presence of a small and fragmented Y chromosomes distributed across the meiotic chromosomes spread. The size of the signal from the probe specific to the target site AgY53B is smaller when compared to probe signal in B and C (white circles). White circles highlight the difference in the sizes of the Y chromosomes between the 3 strains. White circles diameter is 2.5 μm. Green arrowheads indicate fragmented Y chromosome. As shown in S3 Fig, signal from AgY53B probes cover most of the Y chromosome in prophase/metaphase cells. Red = X chromosome specific probes, rDNA locus or conting_240. Green = Y chromosome specific probe, AgY53B. Scale bars 5 μm. (TIF) [file pgen.1011303.s007.tif]
