# Supplementary material for: Y chromosome shredding in Anopheles gambiae: Insight into the cellular dynamics of a novel synthetic sex ratio distorter
Source: PLoS Genet. 2024 Jun 7;20(6):e1011303. doi: 10.1371/journal.pgen.1011303 (PMC11189259; doi:10.1371/journal.pgen.1011303)
Supplement: S7 File — (DOCX) [file pgen.1011303.s014.docx]

LOCUS pBac[AttB-3xP3-RFP-B2- 10968 bp ds-DNA circular 15-APR-2024

DEFINITION .

FEATURES Location/Qualifiers

misc_binding complement(24..850)

/label="piggyBac\Left-3"

/ApEinfo_revcolor="#c6c9d1"

/ApEinfo_fwdcolor="#c6c9d1"

misc_feature 851..906

/label="AttB\Left"

/ApEinfo_revcolor="#84b0dc"

/ApEinfo_fwdcolor="#84b0dc"

terminator complement(921..1150)

/label="SV40\Terminator"

/ApEinfo_revcolor="#f58a5e"

/ApEinfo_fwdcolor="#f58a5e"

CDS complement(1165..1842)

/label="RFP"

/ApEinfo_revcolor="#d59687"

/ApEinfo_fwdcolor="#d59687"

primer_bind 1204..1223

/label="Primer_Bind_1"

/ApEinfo_revcolor="#ff9ccd"

/ApEinfo_fwdcolor="#ff9ccd"

modified_base 1853..1853

/label="Modified_Base_1"

/ApEinfo_revcolor="#d6b295"

/ApEinfo_fwdcolor="#d6b295"

modified_base 1858..1858

/label="Modified_Base_2"

/ApEinfo_revcolor="#c6c9d1"

/ApEinfo_fwdcolor="#c6c9d1"

promoter complement(1878..2128)

/label="3xP3\Promoter"

/ApEinfo_revcolor="#b1ff67"

/ApEinfo_fwdcolor="#b1ff67"

promoter 2135..2633

/label="b2\promoter"

/ApEinfo_revcolor="#b4abac"

/ApEinfo_fwdcolor="#b4abac"

sig_peptide 2640..2708

/label="3xFLAG"

/ApEinfo_revcolor="#f8d3a9"

/ApEinfo_fwdcolor="#f8d3a9"

misc_feature 2640..6908

/label="hCas9\(+\3xFLAG\and\2xNLS)"

/ApEinfo_revcolor="#d6b295"

/ApEinfo_fwdcolor="#d6b295"

sig_peptide 2709..2759

/label="NLS"

/ApEinfo_revcolor="#75c6a9"

/ApEinfo_fwdcolor="#75c6a9"

CDS 2760..6860

/label="hCas9"

/ApEinfo_revcolor="#c6c9d1"

/ApEinfo_fwdcolor="#c6c9d1"

misc_feature 6861..6908

/label="NLS"

/ApEinfo_revcolor="#f8d3a9"

/ApEinfo_fwdcolor="#f8d3a9"

misc_feature 6920..7406

/label="b2\3'UTR"

/ApEinfo_revcolor="#faac61"

/ApEinfo_fwdcolor="#faac61"

promoter 7415..7557

/label="U6\Promoter"

/ApEinfo_revcolor="#ffef86"

/ApEinfo_fwdcolor="#ffef86"

misc_feature 7554..7557

/label="OVH"

/ApEinfo_revcolor="#9eafd2"

/ApEinfo_fwdcolor="#9eafd2"

misc_feature 7558..7575

/label="AgY53B"

/ApEinfo_revcolor="#f8d3a9"

/ApEinfo_fwdcolor="#f8d3a9"

misc_feature 7576..7579

/label="OVH"

/ApEinfo_revcolor="#9eafd2"

/ApEinfo_fwdcolor="#9eafd2"

misc_RNA 7576..7652

/label="gRNA\Backbone"

/ApEinfo_revcolor="#b7e6d7"

/ApEinfo_fwdcolor="#b7e6d7"

terminator 7652..7659

/label="U6\Terminator"

/ApEinfo_revcolor="#84b0dc"

/ApEinfo_fwdcolor="#84b0dc"

misc_recomb 7673..7728

/label="attB\Right"

/ApEinfo_revcolor="#d6b295"

/ApEinfo_fwdcolor="#d6b295"

misc_binding 7729..8429

/label="piggyBac\Left-5"

/ApEinfo_revcolor="#d6b295"

/ApEinfo_fwdcolor="#d6b295"

misc_feature complement(9171..9843)

/label="Ori"

/ApEinfo_revcolor="#84b0dc"

/ApEinfo_fwdcolor="#84b0dc"

CDS complement(9941..10600)

/label="Chloramphenicol-R"

/ApEinfo_revcolor="#ff9ccd"

/ApEinfo_fwdcolor="#ff9ccd"

ORIGIN

1 aagtatagga acttctgaag tggggtattc acgacagcag gctgaataat aaaaaaatta

61 gaaactatta tttaacccta gaaagataat catattgtga cgtacgttaa agataatcat

121 gcgtaaaatt gacgcatgtg ttttatcggt ctgtatatcg aggtttattt attaatttga

181 atagatatta agttttatta tatttacact tacatactaa taataaattc aacaaacaat

241 ttatttatgt ttatttattt attaaaaaaa aacaaaaact caaaatttct tctataaagt

301 aacaaaactt ttaaacattc tctcttttac aaaaataaac ttattttgta ctttaaaaac

361 agtcatgttg tattataaaa taagtaatta gcttaactta tacataatag aaacaaatta

421 tacttattag tcagtcagaa acaactttgg cacatatcaa tattatgctc tcgacaaata

481 acttttttgc attttttgca cgatgcattt gcctttcgcc ttattttaga ggggcagtaa

541 gtacagtaag tacgtttttt cattactggc tcttcagtac tgtcatctga tgtaccaggc

601 acttcatttg gcaaaatatt agagatatta tcgcgcaaat atctcttcaa agtaggagct

661 tctaaacgct tacgcataaa cgatgacgtc aggctcatgt aaaggtttct cataaatttt

721 ttgcgacttt gaaccttttc tcccttgcta ctgacattat ggctgtatat aataaaagaa

781 tttatgcagg caatgtttat cattccgtac aataatgcca taggccacct attcgtcttc

841 ctactgcagg tgcgggtgcc agggcgtgcc cttgggctcc ccgggcgcgt actccacctc

901 acccatgcga tcgctccgga aagatacatt gatgagtttg gacaaaccac aactagaatg

961 cagtgaaaaa aatgctttat ttgtgaaatt tgtgatgcta ttgctttatt tgtaaccatt

1021 ataagctgca ataaacaagt taacaacaac aattgcattc attttatgtt tcaggttcag

1081 ggggaggtgt gggaggtttt ttaaagcaag taaaacctct acaaatgtgg tatggctgat

1141 tatgatctag agtcgcggcc gctacaggaa caggtggtgg cggccctcgg tgcgctcgta

1201 ctgctccacg atggtgtagt cctcgttgtg ggaggtgatg tccagcttgg agtccacgta

1261 gtagtagccg ggcagctgca cgggcttctt ggccatgtag atggacttga actccaccag

1321 gtagtggccg ccgtccttca gcttcagggc cttgtggatc tcgcccttca gcacgccgtc

1381 gcgggggtac aggcgctcgg tggaggcctc ccagcccatg gtcttcttct gcattacggg

1441 gccgtcggag gggaagttca cgccgatgaa cttcaccttg tagatgaagc agccgtcctg

1501 cagggaggag tcttgggtca cggtcaccac gccgccgtcc tcgaagttca tcacgcgctc

1561 ccacttgaag ccctcgggga aggacagctt cttgtagtcg gggatgtcgg cggggtgctt

1621 cacgtacacc ttggagccgt actggaactg gggggacagg atgtcccagg cgaagggcag

1681 ggggccgccc ttggtcacct tcagcttcac ggtgttgtgg ccctcgtagg ggcggccctc

1741 gccctcgccc tcgatctcga actcgtggcc gttcacggtg ccctccatgc gcaccttgaa

1801 gcgcatgaac tccttgatga cgttcttgga ggagcgcacc atggtggcga cctgtgggtc

1861 ccgggcccgc ggtaccgtcg actctagcgg taccccgatt gtttagcttg ttcagctgcg

1921 cttgtttatt tgcttagctt tcgcttagcg acgtgttcac tttgcttgtt tgaattgaat

1981 tgtcgctccg tagacgaagc gcctctattt atactccggc ggtcgagggt tcgaaatcga

2041 taagcttgga tcctaattga attagctcta attgaattag tctctaattg aattagatcc

2101 ccgggcgagc tcgaattaac cattgtggac cggtctagcg ttcataattg atatagtttt

2161 gtaaatgaca tgacagtttt ttattttttt tttatccata attacgaatt gaacaactct

2221 acacacatat taattgcaag aacttatgct acataatatg gaggaaagtg gatgcatcat

2281 cccatccaag aagacatacg aatttatttg tggcatcgca atcggccgaa ccagcaaaag

2341 atgatcatag tagtcatatg agccgtacgt gccggatcat ttcgtgcaga accttcagag

2401 acgttggtcg acagattgat agaaactgtg tagttagtcc attcacaaag ttgttccatt

2461 agggacaaaa gaaaaaaacg gcttaaacta gaaatttgtg tacccagtag gaatcgcgta

2521 ttcggccgat agaggtcctt ccgtaagtat tcccagcgct tagagagcaa cgctcgtgcg

2581 ttccaaaatc cgctaaatat caaacggctt tcacagtttc gaaagatatc aagctcgaca

2641 tggactataa ggaccacgac ggagactaca aggatcatga tattgattac aaagacgatg

2701 acgataagat ggccccaaag aagaagcgga aggtcggtat ccacggagtc ccagcagccg

2761 acaagaagta cagcatcggc ctggacatcg gcaccaactc tgtgggctgg gccgtgatca

2821 ccgacgagta caaggtgccc agcaagaaat tcaaggtgct gggcaacacc gaccggcaca

2881 gcatcaagaa gaacctgatc ggagccctgc tgttcgacag cggcgaaaca gccgaggcca

2941 cccggctgaa gagaaccgcc agaagaagat acaccagacg gaagaaccgg atctgctatc

3001 tgcaagagat cttcagcaac gagatggcca aggtggacga cagcttcttc cacagactgg

3061 aagagtcctt cctggtggaa gaggataaga agcacgagcg gcaccccatc ttcggcaaca

3121 tcgtggacga ggtggcctac cacgagaagt accccaccat ctaccacctg agaaagaaac

3181 tggtggacag caccgacaag gccgacctgc ggctgatcta tctggccctg gcccacatga

3241 tcaagttccg gggccacttc ctgatcgagg gcgacctgaa ccccgacaac agcgacgtgg

3301 acaagctgtt catccagctg gtgcagacct acaaccagct gttcgaggaa aaccccatca

3361 acgccagcgg cgtggacgcc aaggccatcc tgtctgccag actgagcaag agcagacggc

3421 tggaaaatct gatcgcccag ctgcccggcg agaagaagaa tggcctgttc ggaaacctga

3481 ttgccctgag cctgggcctg acccccaact tcaagagcaa cttcgacctg gccgaggatg

3541 ccaaactgca gctgagcaag gacacctacg acgacgacct ggacaacctg ctggcccaga

3601 tcggcgacca gtacgccgac ctgtttctgg ccgccaagaa cctgtccgac gccatcctgc

3661 tgagcgacat cctgagagtg aacaccgaga tcaccaaggc ccccctgagc gcctctatga

3721 tcaagagata cgacgagcac caccaggacc tgaccctgct gaaagctctc gtgcggcagc

3781 agctgcctga gaagtacaaa gagattttct tcgaccagag caagaacggc tacgccggct

3841 acattgacgg cggagccagc caggaagagt tctacaagtt catcaagccc atcctggaaa

3901 agatggacgg caccgaggaa ctgctcgtga agctgaacag agaggacctg ctgcggaagc

3961 agcggacctt cgacaacggc agcatccccc accagatcca cctgggagag ctgcacgcca

4021 ttctgcggcg gcaggaagat ttttacccat tcctgaagga caaccgggaa aagatcgaga

4081 agatcctgac cttccgcatc ccctactacg tgggccctct ggccagggga aacagcagat

4141 tcgcctggat gaccagaaag agcgaggaaa ccatcacccc ctggaacttc gaggaagtgg

4201 tggacaaggg cgcttccgcc cagagcttca tcgagcggat gaccaacttc gataagaacc

4261 tgcccaacga gaaggtgctg cccaagcaca gcctgctgta cgagtacttc accgtgtata

4321 acgagctgac caaagtgaaa tacgtgaccg agggaatgag aaagcccgcc ttcctgagcg

4381 gcgagcagaa aaaggccatc gtggacctgc tgttcaagac caaccggaaa gtgaccgtga

4441 agcagctgaa agaggactac ttcaagaaaa tcgagtgctt cgactccgtg gaaatctccg

4501 gcgtggaaga tcggttcaac gcctccctgg gcacatacca cgatctgctg aaaattatca

4561 aggacaagga cttcctggac aatgaggaaa acgaggacat tctggaagat atcgtgctga

4621 ccctgacact gtttgaggac agagagatga tcgaggaacg gctgaaaacc tatgcccacc

4681 tgttcgacga caaagtgatg aagcagctga agcggcggag atacaccggc tggggcaggc

4741 tgagccggaa gctgatcaac ggcatccggg acaagcagtc cggcaagaca atcctggatt

4801 tcctgaagtc cgacggcttc gccaacagaa acttcatgca gctgatccac gacgacagcc

4861 tgacctttaa agaggacatc cagaaagccc aggtgtccgg ccagggcgat agcctgcacg

4921 agcacattgc caatctggcc ggcagccccg ccattaagaa gggcatcctg cagacagtga

4981 aggtggtgga cgagctcgtg aaagtgatgg gccggcacaa gcccgagaac atcgtgatcg

5041 aaatggccag agagaaccag accacccaga agggacagaa gaacagccgc gagagaatga

5101 agcggatcga agagggcatc aaagagctgg gcagccagat cctgaaagaa caccccgtgg

5161 aaaacaccca gctgcagaac gagaagctgt acctgtacta cctgcagaat gggcgggata

5221 tgtacgtgga ccaggaactg gacatcaacc ggctgtccga ctacgatgtg gaccatatcg

5281 tgcctcagag ctttctgaag gacgactcca tcgacaacaa ggtgctgacc agaagcgaca

5341 agaaccgggg caagagcgac aacgtgccct ccgaagaggt cgtgaagaag atgaagaact

5401 actggcggca gctgctgaac gccaagctga ttacccagag aaagttcgac aatctgacca

5461 aggccgagag aggcggcctg agcgaactgg ataaggccgg cttcatcaag agacagctgg

5521 tggaaacccg gcagatcaca aagcacgtgg cacagatcct ggactcccgg atgaacacta

5581 agtacgacga gaatgacaag ctgatccggg aagtgaaagt gatcaccctg aagtccaagc

5641 tggtgtccga tttccggaag gatttccagt tttacaaagt gcgcgagatc aacaactacc

5701 accacgccca cgacgcctac ctgaacgccg tcgtgggaac cgccctgatc aaaaagtacc

5761 ctaagctgga aagcgagttc gtgtacggcg actacaaggt gtacgacgtg cggaagatga

5821 tcgccaagag cgagcaggaa atcggcaagg ctaccgccaa gtacttcttc tacagcaaca

5881 tcatgaactt tttcaagacc gagattaccc tggccaacgg cgagatccgg aagcggcctc

5941 tgatcgagac aaacggcgaa accggggaga tcgtgtggga taagggccgg gattttgcca

6001 ccgtgcggaa agtgctgagc atgccccaag tgaatatcgt gaaaaagacc gaggtgcaga

6061 caggcggctt cagcaaagag tctatcctgc ccaagaggaa cagcgataag ctgatcgcca

6121 gaaagaagga ctgggaccct aagaagtacg gcggcttcga cagccccacc gtggcctatt

6181 ctgtgctggt ggtggccaaa gtggaaaagg gcaagtccaa gaaactgaag agtgtgaaag

6241 agctgctggg gatcaccatc atggaaagaa gcagcttcga gaagaatccc atcgactttc

6301 tggaagccaa gggctacaaa gaagtgaaaa aggacctgat catcaagctg cctaagtact

6361 ccctgttcga gctggaaaac ggccggaaga gaatgctggc ctctgccggc gaactgcaga

6421 agggaaacga actggccctg ccctccaaat atgtgaactt cctgtacctg gccagccact

6481 atgagaagct gaagggctcc cccgaggata atgagcagaa acagctgttt gtggaacagc

6541 acaagcacta cctggacgag atcatcgagc agatcagcga gttctccaag agagtgatcc

6601 tggccgacgc taatctggac aaagtgctgt ccgcctacaa caagcaccgg gataagccca

6661 tcagagagca ggccgagaat atcatccacc tgtttaccct gaccaatctg ggagcccctg

6721 ccgccttcaa gtactttgac accaccatcg accggaagag gtacaccagc accaaagagg

6781 tgctggacgc caccctgatc caccagagca tcaccggcct gtacgagaca cggatcgacc

6841 tgtctcagct gggaggcgac aaaaggccgg cggccacgaa aaaggccggc caggcaaaaa

6901 agaaaaagta attaattaac taaagctaaa ttgaacaccc taaattatgt gtaaaatttc

6961 tgctaagcag cggtgttggg gtcaataaaa atgttttttt ccactctatt cgcttcgttt

7021 ttgttgccat ttctcagttt ttgcttcgta ctcatgtgta aggattagtg cagtgatggg

7081 aagtagctcc gaagttttct ggaatcgttt ccggatagta ggttcggtat tagtttccgg

7141 aatcggctcc ggaattggtt ccggaattga ttccgggatc agaattggct caaaattctc

7201 atggagattc ccagagtgat ttcgcttctg aaacttcgta tttaattcaa gaattgatcc

7261 ccattctgga gctaattcca attctggagt caattctgat tctgttaccc gaacaaattg

7321 cgattcccag gtcaatgccc attccgggga cgattctgat tccggagttg gaatcagctc

7381 cggaattgga atcggtcctt aaatcgggcg cgcctttgta tgcgtgcgct tgaagggttg

7441 atcggaacct tacaacagtt gtagctatac ggctgcgtgt ggcttctaac gttatccatc

7501 gctagaagtg aaacgaatgt gcgtaggtat atatatgaaa tggagttgct ctctgctGAA

7561 TAGAATCAGA AAAGTgtttt agagctagaa atagcaagtt aaaataaggc tagtccgtta

7621 tcaacttgaa aaagtggcac cgagtcggtg ctttttttta cgcgtgggtc ccatgggtga

7681 ggtggagtac gcgcccgggg agcccaaggg cacgccctgg cacccgcaga tctcggatct

7741 gacaatgttc agtgcagaga ctcggctacg cctcgtggac tttgaagttg accaacaatg

7801 tttattctta cctctaatag tcctctgtgg caaggtcaag attctgttag aagccaatga

7861 agaacctggt tgttcaataa cattttgttc gtctaatatt tcactaccgc ttgacgttgg

7921 ctgcacttca tgtacctcat ctataaacgc ttcttctgta tcgctctgga cgtcatcttc

7981 acttacgtga tctgatattt cactgtcaga atcctcacca acaagctcgt catcgctttg

8041 cagaagagca gagaggatat gctcatcgtc taaagaacta cccattttat tatatattag

8101 tcacgatatc tataacaaga aaatatatat ataataagtt atcacgtaag tagaacatga

8161 aataacaata taattatcgt atgagttaaa tcttaaaagt cacgtaaaag ataatcatgc

8221 gtcattttga ctcacgcggt cgttatagtt caaaatcagt gacacttacc gcattgacaa

8281 gcacgcctca cgggagctcc aagcggcgac tgagatgtcc taaatgcaca gcgacggatt

8341 cgcgctattt agaaagagag agcaatattt caagaatgca tgcgtcaatt ttacgcagac

8401 tatctttcta gggttaaaaa agatttgcga aaatgaagtg aagttcctat actttctaga

8461 gaataggaac ttctatagtg agtcgaataa gggcgacaca aaatttattc taaatgcata

8521 ataaatactg ataacatctt atagtttgta ttatattttg tattatcgtt gacatgtata

8581 attttgatat caaaaactga ttttcccttt attattttcg agatttattt tcttaattct

8641 ctttaacaaa ctagaaatat tgtatataca aaaaatcata aataatagat gaatagttta

8701 attataggtg ttcatcaatc gaaaaagcaa cgtatcttat ttaaagtgcg ttgctttttt

8761 ctcatttata aggttaaata attctcatat atcaagcaaa gtgacaggcg cccttaaata

8821 ttctgacaaa tgctctttcc ctaaactccc cccataaaaa aacccgccga agcgggtttt

8881 tacgttattt gcggattaac gattactcgt tatcagaacc gcccaggggg cccgagctta

8941 agactggccg tcgttttaca acacagaaag agtttgtaga aacgcaaaaa ggccatccgt

9001 caggggcctt ctgcttagtt tgatgcctgg cagttcccta ctctcgcctt ccgcttcctc

9061 gctcactgac tcgctgcgct cggtcgttcg gctgcggcga gcggtatcag ctcactcaaa

9121 ggcggtaata cggttatcca cagaatcagg ggataacgca ggaaagaaca tgtgagcaaa

9181 aggccagcaa aaggccagga accgtaaaaa ggccgcgttg ctggcgtttt tccataggct

9241 ccgcccccct gacgagcatc acaaaaatcg acgctcaagt cagaggtggc gaaacccgac

9301 aggactataa agataccagg cgtttccccc tggaagctcc ctcgtgcgct ctcctgttcc

9361 gaccctgccg cttaccggat acctgtccgc ctttctccct tcgggaagcg tggcgctttc

9421 tcatagctca cgctgtaggt atctcagttc ggtgtaggtc gttcgctcca agctgggctg

9481 tgtgcacgaa ccccccgttc agcccgaccg ctgcgcctta tccggtaact atcgtcttga

9541 gtccaacccg gtaagacacg acttatcgcc actggcagca gccactggta acaggattag

9601 cagagcgagg tatgtaggcg gtgctacaga gttcttgaag tggtgggcta actacggcta

9661 cactagaaga acagtatttg gtatctgcgc tctgctgaag ccagttacct tcggaaaaag

9721 agttggtagc tcttgatccg gcaaacaaac caccgctggt agcggtggtt tttttgtttg

9781 caagcagcag attacgcgca gaaaaaaagg atctcaagaa gatcctttga tcttttctac

9841 ggggtctgac gctcagtgga acgacgcgcg cgtaactcac gttaagggat tttggtcatg

9901 agcttgcgcc gtcccgtcaa gtcagcgtat tttcgagacg ttacgccccg ccctgccact

9961 catcgcagta ctgttgtaat tcattaagca ttctgccgac atggaagcca tcacaaacgg

10021 catgatgaac ctgaatcgcc agcggcatca gcaccttgtc gccttgcgta taatatttgc

10081 ccatggtgaa aacgggggcg aagaagttgt ccatattggc cacgtttaaa tcaaaactgg

10141 tgaaactcac ccagggattg gctgacacga aaaacatatt ctcaataaat cctttaggga

10201 aataggccag gttttcaccg taacacgcca catcttgcga atatatgtgt agaaactgcc

10261 ggaaatcgtc gtggtattca ctccagagcg atgaaaacgt ttcagtttgc tcatggaaaa

10321 cggtgtaaca tgggtgaaca ctatcccata tcaccagctc accgtctttc attgccatac

10381 ggaattctgg atgagcattc atcaggcggg caagaatgtg aataaaggcc ggataaaact

10441 tgtgcttatt tttctttacg gtttttaaaa aggccgtaat atccagctga acggtctggt

10501 tataggtaca ttgagcaact gactgaaatg cctcaaaatg ttctttacga tgccattggg

10561 atatatcaac ggtggtatat ccagtgattt ttttctccat attcttcctt tttcaatatt

10621 attgaagcat ttatcagggt tattgtctca tgagcggata catatttgaa tgtatttaga

10681 aaaataaaca aataggggtc agtgttacaa ccaattaacc aattctgatg cgcgtctctc

10741 ccctttgcct ggcggcagta gcgcggtggt cccacctgac cccatgccga actcagaagt

10801 gaaacgccgt agcgccgatg gtagtgtggg gactccccat gcgagagtag ggaactgcca

10861 ggcatcaaat aaaacgaaag gctcagtcga aagactgggc ctttcgcccg ggctaattag

10921 ggggtgtcgc ccttattcga ctctatagtg aagttcctat tctctaga

//
